# Supplementary material for: Understanding the biological processes of kidney carcinogenesis: an integrative multi-omics approach
Source: Mol Syst Biol. 2024 Nov 26;20(12):1282–302. doi: 10.1038/s44320-024-00072-3 (PMC11612429; doi:10.1038/s44320-024-00072-3)
Supplement: Supplementary file 1 — Appendix [file 44320_2024_72_MOESM1_ESM.pdf]

Appendix for Understanding the biological processes of kidney carcinogenesis: an integrative multi-omics approach

Table of Content

Appendix Figure S1..Page 2

Appendix Figure S2..Page 3

Appendix Figure S3..Page 4

Appendix Figure S4..Page 5

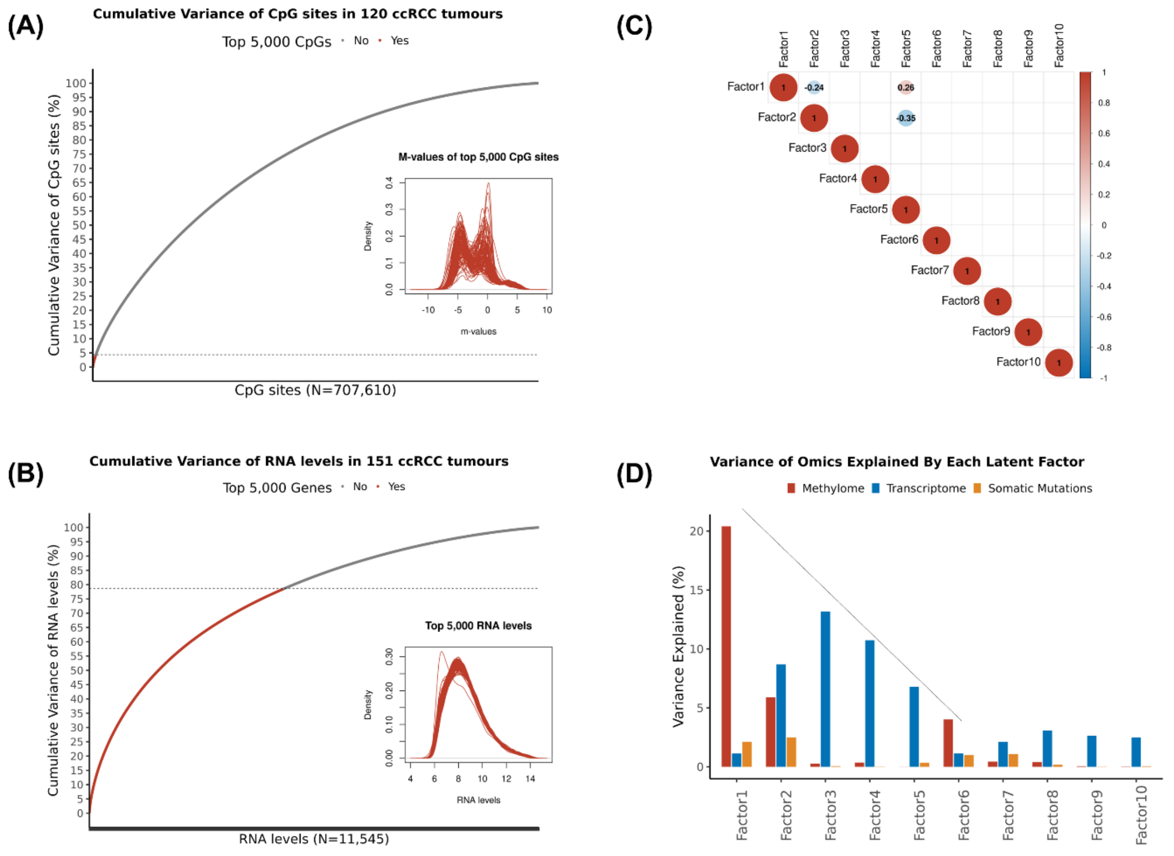

Appendix Figure S1. Description of MOFA. (A-B) The features by omic layer were ranked according to variance across samples (left to right) and cumulative variance was calculated. Dashed line represents the respective cumulative variance threshold by the features included (red) or not (grey) as MOFA inputs. Of the features included in MOFA, 5% and 78% of cumulative variance was observed across samples in DNA methylation and RNA levels (transcriptome), respectively. The distributions of the 5,000 most variable features were plotted. (C) Correlation matrix between the 10 LF generated by MOFA. Empty boxes when non-adjusted p-values > 0.05. Positive correlations in blue, negative in red. (D) Percentage of variance in each omic layer (methylome in red, transcriptome in blue, and somatic mutation profile in yellow) explained by LF. The elbow shape on the curve represented as solid black line.

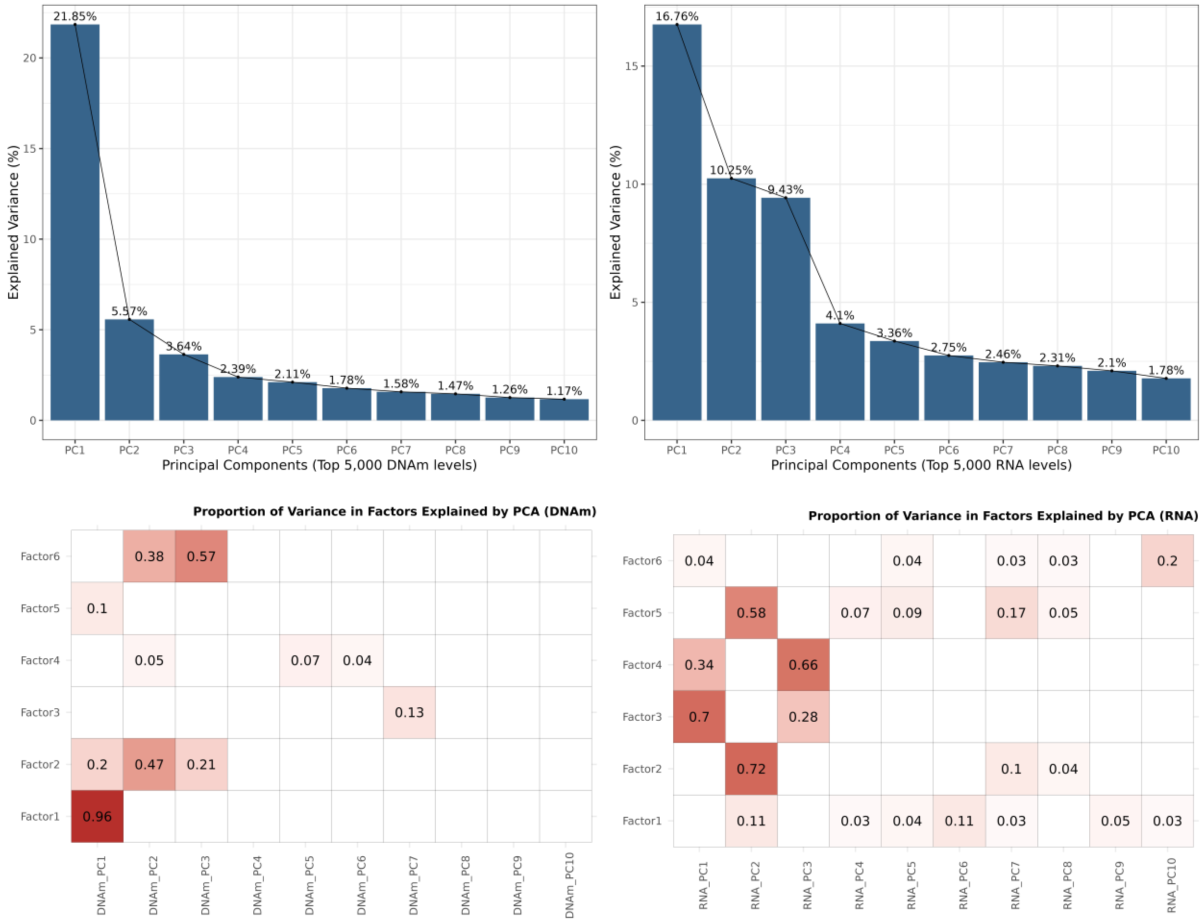

Appendix Figure S2. Comparison of single-omic with integrative multi-omics approaches. Principal component analysis was performed using the same top 5,000 features in each omic layer as those included in the integrative multi-omics approach. The variance across samples explained by each component (PC1-10) was represented in the top left bar plot (DNA methylation data) and in the top right (transcriptome data). Direct comparison between latent factors (Factor1-6) and principal components from DNA methylation (DNAm_PC; bottom left) or transcriptome data (RNA_PC; bottom right). The results were represented as R^2 by exponentiating the pairwise Pearson's correlation coefficients. Comparisons with Pearson's correlation p<0.05 were represented.

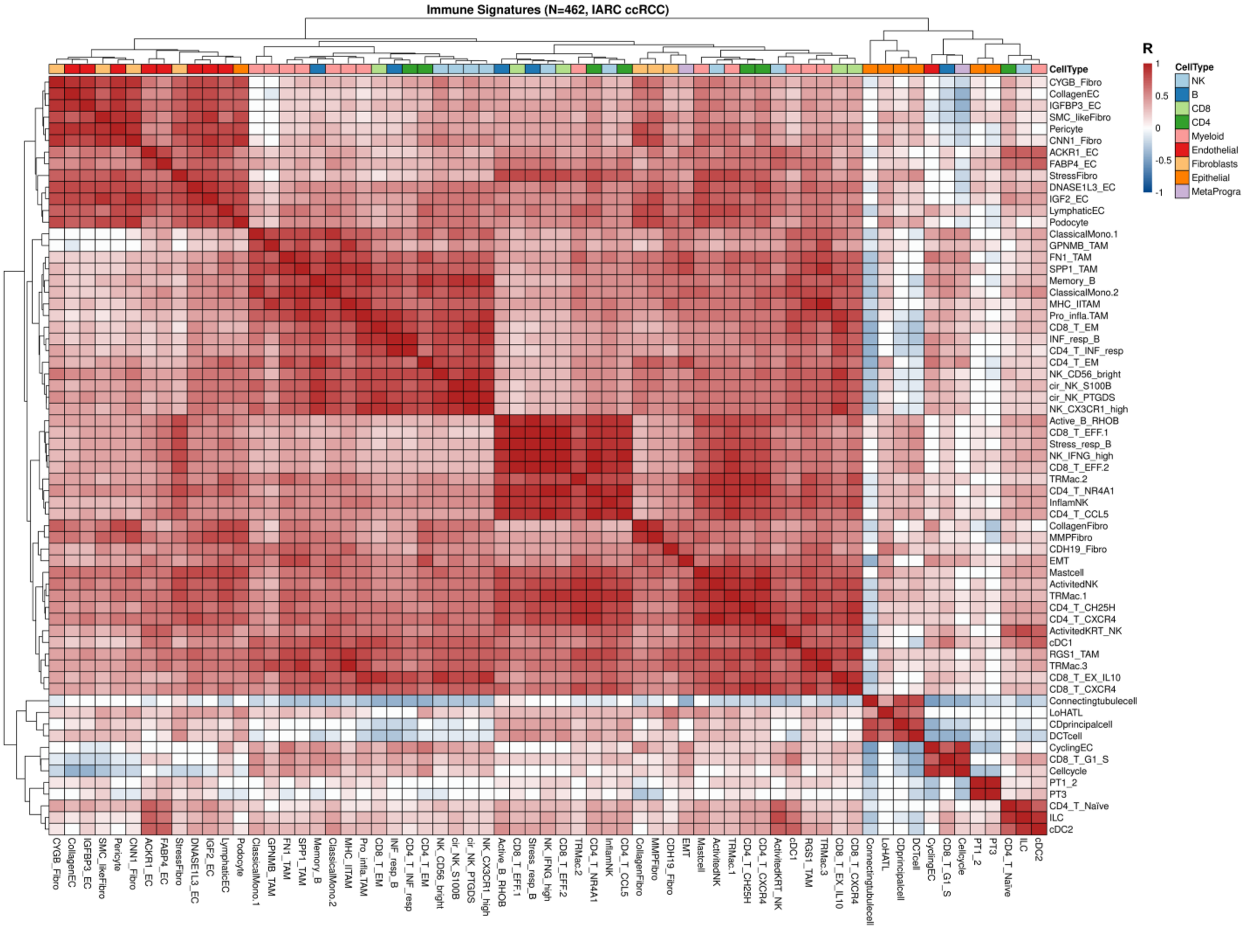

Appendix Figure S3. Correlation of tumour microenvironment signatures in ccRCC bulk tumour transcriptome. Pearson's correlation matrix of the 65 gene expression signatures derived from single-cell RNA sequencing (Li *et al.*, 2022) in ccRCC tumours from IARC ccRCC validation series (N=462), generated using pheatmap R package. Hierarchical clustering of signatures was performed, and individual cell types are coloured in the legend by family membership (NK, natural killer cells; CD8, CD8+ T cells; CD4, CD4+ T cells; Myeloid cells; Endothelial cells; Fibroblasts; Epithelial cells; MetaProgram, ccRCC meta-program). Positive correlations in shades of red, negative ones in shades of blue.

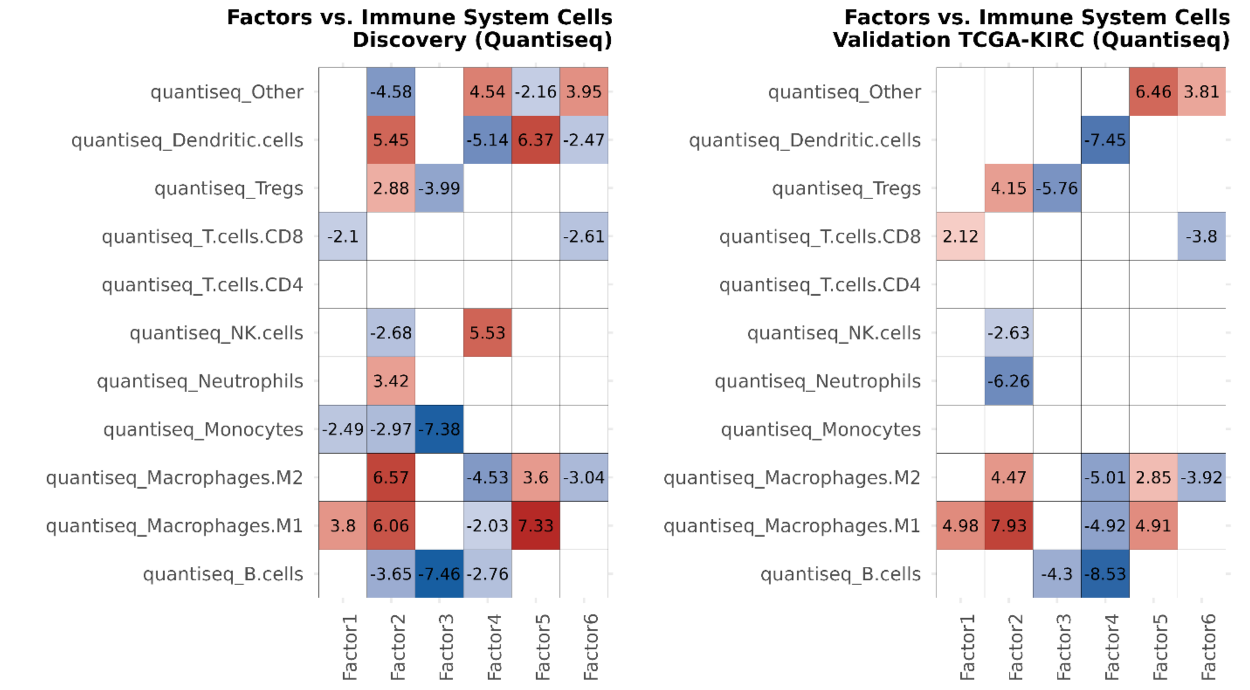

Appendix Figure S4. Deconvolution analysis of immune cells in ccRCC tumours. Linear regression analyses were used to evaluate the associations between the proportions of immune cells defined using the deconvolution analysis (quantiseq package in R) and latent factors in the discovery (left) and validation (right) sets. The associations were represented as Z-scores (beta estimates divided by the standard errors). Positive associations (Z-score>0). Negative associations (Z-score<0). Blank squares when associations displayed p>0.05. The linear regression model was: Latent factor ~ Immune cell proportion + age at diagnosis + sex.
