# Supplementary material for: Understanding the biological processes of kidney carcinogenesis: an integrative multi-omics approach
Source: Mol Syst Biol. 2024 Nov 26;20(12):1282–302. doi: 10.1038/s44320-024-00072-3 (PMC11612429; doi:10.1038/s44320-024-00072-3)
Supplement: Supplementary file 20 — Expanded View Figures [file 44320_2024_72_MOESM20_ESM.pdf]

Expanded View Figures

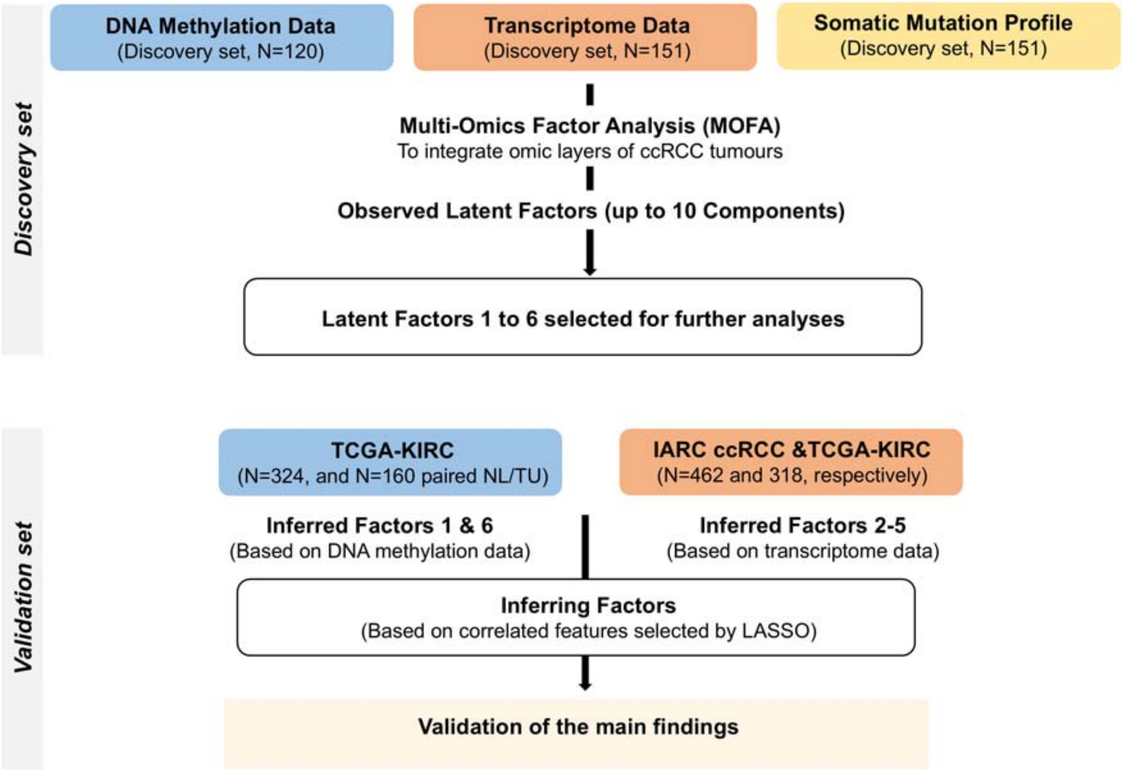

Figure EV1. Study design.

(Upper) For the discovery set (IARC series), we used DNA methylation (120 tumours), transcriptome (151 tumours), and somatic mutation profile derived from whole-genome sequencing data of the overlapping clear cell renal cell carcinoma (ccRCC) tumour samples (151 tumours) to perform a Multi-Omics Factor Analysis (MOFA) to uncover the sources of inter-patient variation in the ccRCC data. For these analyses, the top 5000 most variable features within DNA methylation (continuous variables; M-values) and transcriptome data (continuous variables; log2-transcripts per million) along with DNA mutational signatures and cancer driver mutations (binary variables; presence or absence) derived from whole-genome sequencing were used as inputs to MOFA. The output of MOFA was 10 orthogonal latent factors. The first six latent factors were selected using the elbow method. In parallel, no additional associations between latent factors 7 to 10 and epidemiological data were observed (more details in Table EV5). (Bottom) For validation purposes, we used Least Absolute Shrinkage and Selection Operator (LASSO) regression models to select the most informative independent features correlated with each latent factor, CpG sites or gene expression levels since these two omics layers accounted for over 90% of inter-patient's variability in the discovery set, and based on them, we calculated signatures to infer the latent factors in tumour and paired normal adjacent/tumour samples in two independent datasets (TCGA-KIRC: 324 and 160 normal-tumour pairs; IARC ccRCC series: 462 tumours).

© World Health Organization

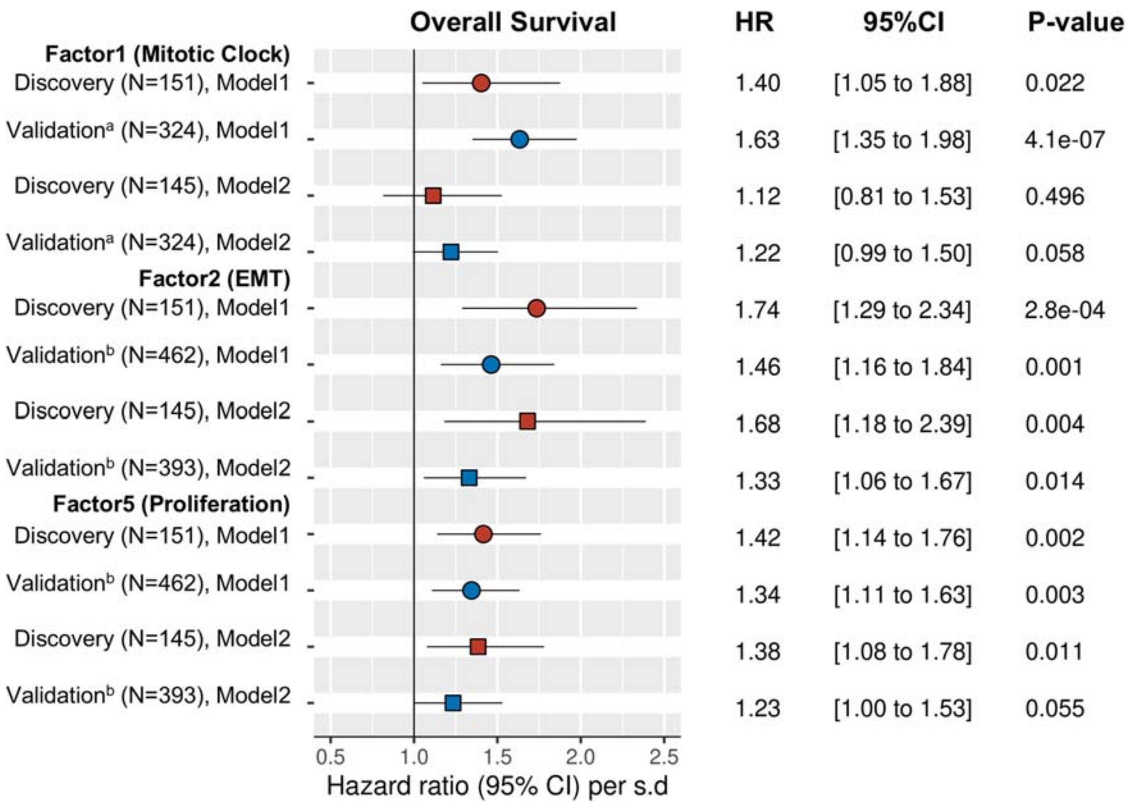

Figure EV2. Molecular components associated with prognosis of ccRCC patients.

Cox proportional-hazards models for assessing overall survival of ccRCC patients in relation to latent factor 1 (mitotic-like epigenetic clock epiTOC2), 2 (epithelial–mesenchymal transition/EMT), and 5 (cell cycle), adjusting for age at diagnosis, sex (model 1; circle shape), and additionally by tumour stage and grade (I + II vs. III + IV; model 2; square shape) in the discovery (red) and validation (blue) datasets. Hazard ratios (HR) represented as an increase in relative mortality risk per 1 unit of standard deviation increase in factors. Two different validation sets were used according to the factors, TCGA-KIRC (a; 324 kidney tumour samples) for latent factor 1 and IARC series sets (b; 462 kidney tumour samples) for latent factors 2 and 5. Observed latent factors used for the regression models in the discovery set while signatures for the same latent factors were used for the analyses in the validation sets. *P* values < 0.05, derived from Cox proportional-hazards models, were considered statistically significant.

 © World Health Organization

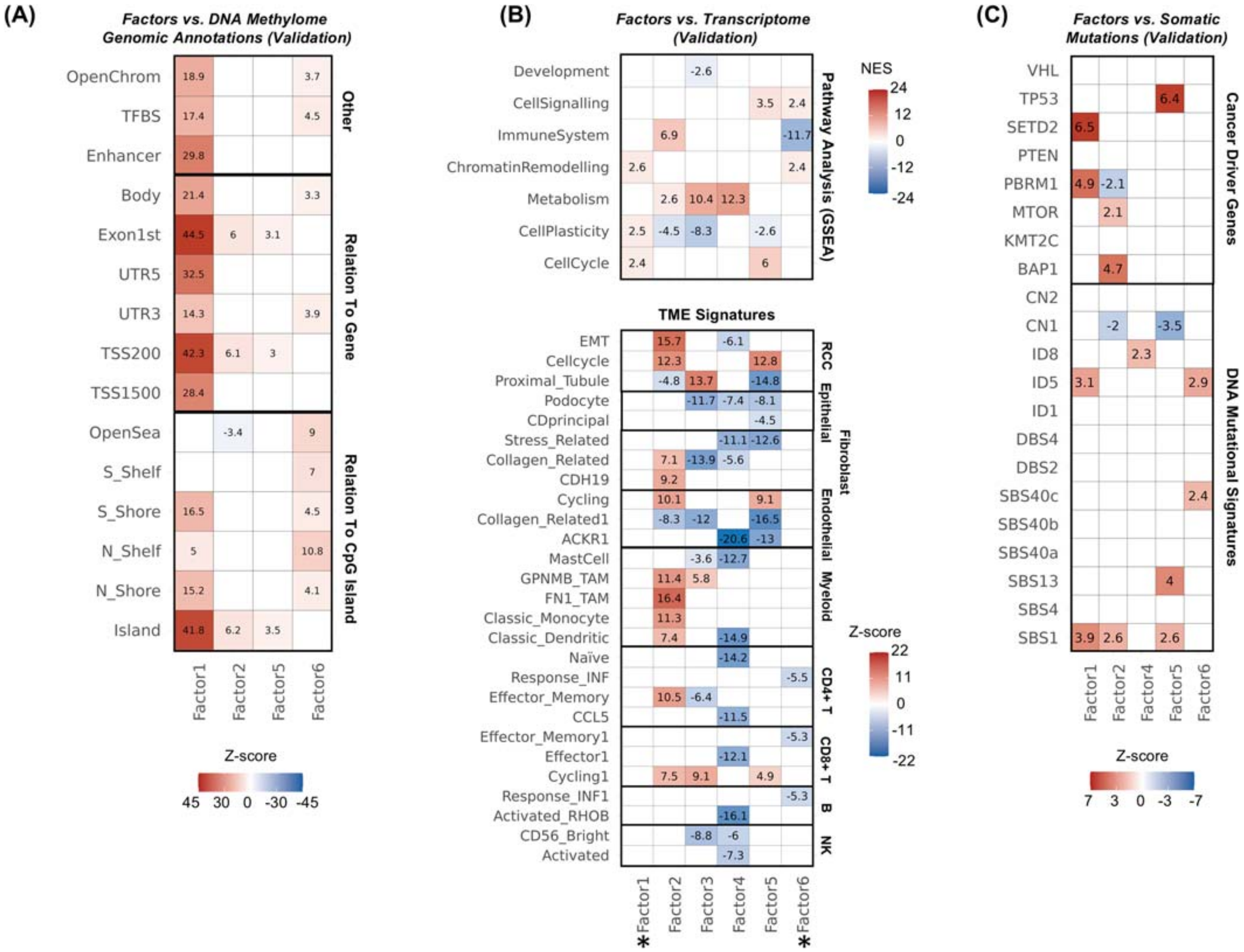

Figure EV3. Validation of the associations between latent factors and molecular features of ccRCC tumours in the independent ccRCC datasets.

For validation purposes, only the statistically significant associations in the discovery set were considered in the validation phase. Heatmaps showing the Z-scores (beta divided by standard error) of linear regression analyses between latent factor signatures (outcome) and genomic annotations and features related to the three omics layers, adjusting by age at diagnosis and sex. (**A**) For the DNA methylome layer (TCGA-KIRC: 324 tumours for DNA methylation-related latent factors 1 and 6; and 323 cases for the expression-related ones, latent factors 2–5), the average beta methylation levels of the 4,000 CpG sites that overlapped with discovery set were summarised by genomic annotations related to CpG island (island, shores, shelves, and open sea), gene (proximal/TSS200 and distal/TSS1500 promoters, UTRs, exons, and body), and other regulatory regions (open chromatin, transcription factor binding site/TFBS, and enhancer) and used as predictors. (**B**) For the transcriptome layer, pathway analysis was performed for the top 500 gene expression levels correlated with each latent factor using the GSEA database and fgsea R package (v1.27.1). Biological pathways present in the discovery set with *P* value < 0.05 were manually categorised into functional groups based on their descriptions in the GSEA database and the canonical functions of the associated genes, including Development, Cell Signalling, Immune System, Chromatin Remodelling, Metabolism, Cell Plasticity, and Cell Cycle. We then summed the normalised enrichment scores (NES), provided by the pathway analysis, by functional category to represent the major biological processes enriched for each latent factor. For the tumour microenvironment (TME) gene expression signatures, it was shown the statistically significant associations between latent factors 1 to 6 and the 27 representative TME signatures in ccRCC tumours. The association estimates were derived from the analyses in the validation sets (IARC ccRCC series: 462 tumours for latent factors 2–5; TCGA-KIRC: 323 tumours for latent factors 1 and 6) after adjustments by covariates (sex, age at diagnosis, and country of origin whenever possible. The associations were represented as Z-scores (beta divided by standard error; Z-scores>0 in shades of red; Z-score<0 in shades of blue). The ccRCC tumour microenvironment signatures (CD4 + T, B, NK, endothelial, myeloid, CD8 + T, epithelial and fibroblast cells) and kidney cancer meta programmes/RCC (epithelial-to-mesenchymal transition/EMT and cell cycle) were derived from previous single-cell RNA sequencing data (Li et al, 2022). (**C**) The somatic profile based on whole-exome sequencing data (TCGA-KIRC: 268 tumours) was represented by ccRCC somatic driver mutations (binary; presence or absence) and DNA mutational signatures (continuous). Regression models included age at diagnosis, sex, and country of origin as covariates. Values represented as shades of red (Z-scores>0) and blue (Z-score<0). The associations with *P* < 0.05, derived from linear regression models, were represented. Tobacco-related (SBS4, DBS2), clock-like (SBS1, ID1), *APOBEC* (SBS13), copy number (CN) and structural variation (SV) DNA mutational signatures. Source data are available online for this figure.

© World Health Organization

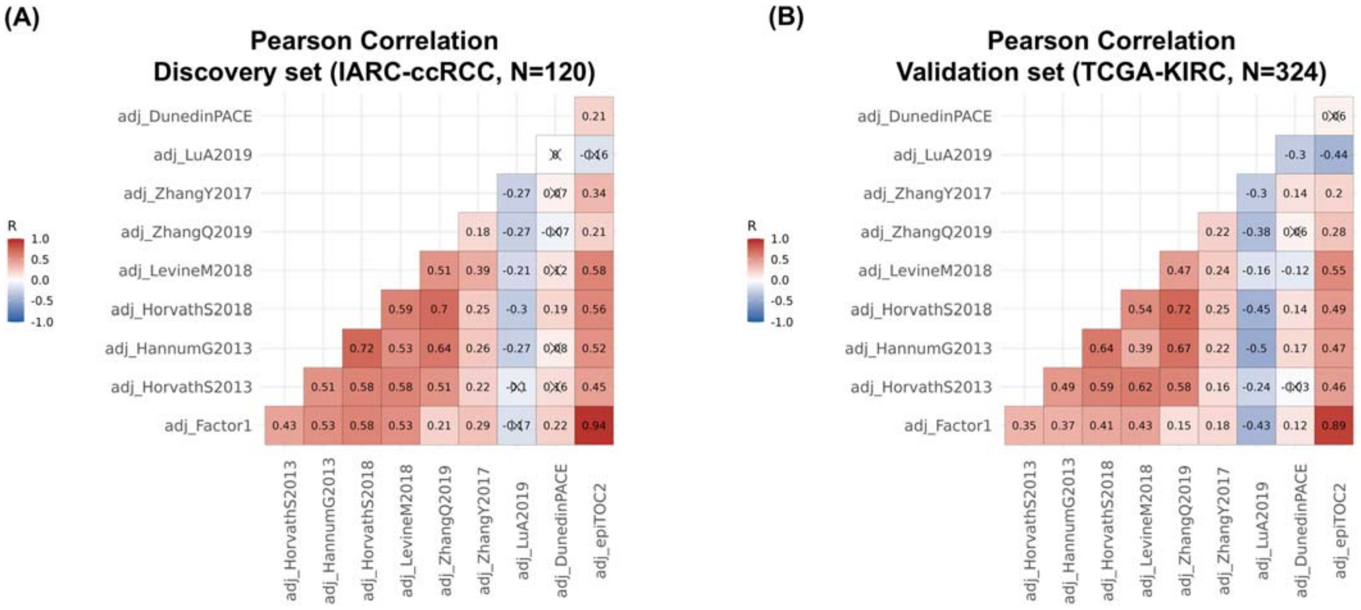

Figure EV4. Correlation between latent factor 1 and epigenetic clocks.

Heatmap represents the Pearson's correlation coefficients between the residuals of epigenetic clocks and latent factor 1 after adjusting by chronological age in ccRCC tumours from (**A**) IARC discovery (120 tumours) and (**B**) validation (TCGA-KIRC: 324 tumours) sets with DNA methylation data. Horvath's (Horvath et al, 2013, Horvath et al, 2018), Hannum's (Hannum et al, 2013), and Zhang's (Zhang et al, 2019) clocks trained on chronological age. EpiTOC2 is designed to predict mitotic cell rate. Zhang's (Zhang et al, 2017), Levine's (Levine et al, 2018), and DunedinPACE (Belsky et al, 2022) clocks to predict mortality risk. Lu's (Lu et al, 2019) to predict telomere length.

 © World Health Organization

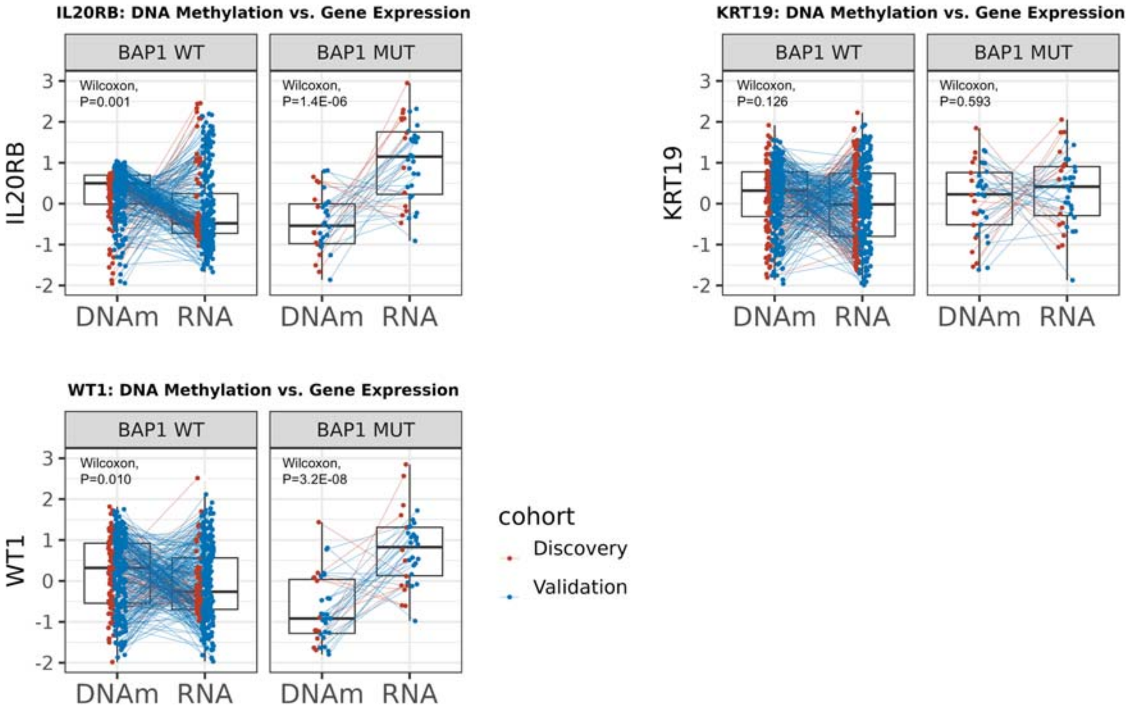

Figure EV5. *BAP1* mutations and the epigenetic regulation of epithelial–mesenchymal transition (EMT) genes in ccRCC tumours.

Comparison of paired DNA methylation (DNAm) and RNA levels of EMT genes (*IL20RB*, *KRT19*, and *WT1*) in ccRCC tumours in the discovery (IARC ccRCC: 120 tumours; lines and dots in red) and validation cohorts (TCGA-KIRC: 268 tumours) by *BAP1* driver mutation status (wild-type: *BAP1* WT or mutated: *BAP1* MUT). Lines connect matched samples. The boxplots depict the distribution of the data with the central line representing the median (50th percentile). The bounds of the box correspond to the interquartile range (IQR), extending from the 25th percentile (lower quartile) to the 75th percentile (upper quartile). The whiskers show the minimum and maximum values within 1.5 times the IQR from the quartiles, while data points beyond the whiskers are considered outliers. *P* values from Wilcoxon signed-rank. *P* values < 0.05 were considered statistically significant.
